# Novel Molecular Mechanisms Underlying Tumorigenesis and Innovative Therapeutic Approaches for Cancer-Fighting

**DOI:** 10.3390/ijms241310956

**Published:** 2023-06-30

**Authors:** Annalisa Pecoraro, Giulia Russo, Annapina Russo

**Affiliations:** Department of Pharmacy, University of Naples “Federico II”, 80131 Naples, Italy; annalisa.pecoraro@unina.it (A.P.); giulia.russo@unina.it (G.R.)

Remarkable advances have been made in cancer therapy; however, the high degree of cellular heterogeneity observed during cancer progression is the major driver in the development of resistant phenotypes upon treatment administration. Tumors are characterized by a mixed population of cells with different molecular features that are responsible for the diverse responsivity to cancer therapy. Thus, a deep understanding of molecular mechanisms underlying tumorigenesis is of fundamental importance in order to design precise and efficient therapies. 

This Special Issue, entitled “Novel Molecular Mechanisms Underlying Tumorigenesis and Innovative Therapeutic Approaches for Cancer-Fighting”, of the *International Journal of Molecular Sciences* includes a total of seven contributions: four original articles and three reviews addressing current knowledge of and progress made in the molecular mechanisms associated with tumorigenesis, and innovative therapeutic approaches developed for cancer therapy.

In the context of innovative therapeutic approaches taking advantage of natural compounds exerting anticancer effects, the potential of physiological methyl donor S-adenosylmethionine (AdoMet) in overcoming drug resistance in colon cancer cells devoid of p53 have been investigated by Mosca et al. [1]. AdoMet is an important and naturally occurring, multifunctional sulfonium compound found in all mammalian cells, in which it exerts a fundamental role in cellular metabolism through different well-documented biological functions.

Specifically, it has been demonstrated that AdoMet-induced antiproliferative effects were correlated to cell cycle arrest in the S-phase, inhibition of autophagy, increased ROS levels, and induction of apoptosis. These findings strongly suggest AdoMet as a potential candidate for novel therapeutic strategies to overcome the drug resistance of colon cancer depending on p53 and ribosomal protein uL3 status. In addition, the synergistic anticancer effect of AdoMet in association with the main chemotherapeutics used in the treatment of breast cancer and head and neck squamous cell carcinoma has been extensively reviewed by Mosca et al. [2]. The authors discussed the promising chemoprotective and synergistic anticancer effects exerted by AdoMet in combination with Doxorubicin and Cisplatin on the main signaling pathways involved in cell death, migration, and invasion processes in comparison with emerging phytochemicals. 

Natural compounds have also shown the potential to affect tumor metastasis and invasion. Jung et al. [3] explored the anticancer effects of matrine (C15H24N2O), a quinolizidine alkaloid isolated from the roots of *Sophora flavescens* and widely used in traditional Chinese herb medicines, in various cancer cell lines. Specifically, the authors focused on its anti-metastasis activities with reference to the CXCR4 (C-X-C chemokine receptor type 4) signaling axis in human lung, prostate, and pancreatic cancer cells. They demonstrated that matrine induced a reduction of CXCR4 and MMP-9/2 (matrix metalloproteinases 9/2) at both mRNA and protein levels, and negatively regulated human HER2 (epidermal growth factor receptor 2) and CXCL12 (C-X-C Motif Chemokine Ligand 12)-induced CXCR4 expression. Finally, NF-kB suppression by matrine caused the inhibition of cancer cells’ metastatic potential. In light of these findings, matrine can be considered a potential candidate for cancer therapy.

Collectively, the studies conducted by Mosca et al. [1] and Jung et al. [3] demonstrated the ability of some natural compounds to target various hallmarks of cancer cells, providing the basis for the development of innovative anticancer therapies. However, further studies will be required to verify the effectiveness of the proposed anticancer agents in in vivo experimental models.

Non-coding RNAs as miRNAs and long non-coding RNAs (lncRNAs) are an appealing molecular class because of their ability to modulate the expression of genes involved in molecular mechanisms orchestrating cancer growth, survival, invasion, and drug resistance. In particular, Barbato et al. [4], used an integrative genomic analysis to investigate the miRNA expression profiles of drug-resistant melanoma patients and cell lines, revealing significant downregulation of microRNA-181a and -181b (miR181a/b). Data showed that miR-181a/b expression restoration was able to reverse the resistance of melanoma cells to the BRAF inhibitor dabrafenib, while miR-181a/b depletion increased resistance in sensitive cell lines. 

These results strongly suggest that the miR-181a/b-TFAM axis plays an important role in the modulation of key components of cancer cell growth in both in vitro and in vivo melanoma models. In this light, miR-181a and -181b may be regarded as novel and potentially potent therapeutic candidates for overcoming resistance in refractory melanomas. 

It is noteworthy that several lncRNAs hinder radiation therapy in esophageal squamous cell carcinoma (ESCC) by regulating the expression of many microRNAs, whereas other lncRNAs sensitize the cancer cells to radiation therapy in ESCC by targeting different cellular pathways. A summary of the current knowledge about the relationship between several lncRNAs and the resistance or sensitivity of ESCC cells to radiotherapy was reported in the review by Sharma et al. [5].

The authors also highlighted possible molecular pathways that regulate radioresistance and sensitivity during ESCC treatment. Overall, the importance of lncRNA-based intervention in deciding treatment modalities for refractory or highly aggressive tumors is emphasized in this review.

Although the RNA molecules discovered by Barbato et al. and Sharma et al. have promising applications in cancer therapy and/or diagnosis, efficient delivery of therapeutic RNA into target tissues and precise detection of RNA markers remain as challenges.

One of the major limitations of current anticancer chemotherapy drugs is that they also target healthy cells, resulting in a variety of side effects. A successful way to improve the target selectivity of anticancer drugs towards tumor cells is reported by Tsafa et al. [6]. They looked into a strategy that combined chemotherapeutic drugs with gene therapy viral vectors in order to improve cancer gene therapy and reduce chemotherapy doses to a less toxic and cost-effective degree. Specifically, the authors designed a hybrid vector (AAVP) containing the recombinant adeno-associated virus genome and a filamentous phage capsid. In addition, on the phage capsid was displayed the double-cyclic CDCRGDCFC (RGD4C) ligand that binds the alpha-V/beta-3 integrin receptor, achieving tumor targeting. Thus, they combined RGD4C/AAVP with doxorubicin and tested its efficacy in cancer cells of human and murine origin. Using a three-dimensional (3D) multicellular tumor spheroid model, they revealed that doxorubicin enhanced gene delivery by RGD4C/AAVP without affecting the vector selectivity for cancer cells, consequently improving cancer cell death. These findings clearly indicated that RGD4C/AAVP could be given safely and weekly in combination with doxorubicin, reducing its potential toxicity; furthermore, this therapeutic approach may not require adjustment of the doxorubicin dosage or the interval between doxorubicin administrations.

The crucial role of autophagy in the regulation of integrated stress responses, including nucleolar and endoplasmic reticulum is discussed in the review Pecoraro et al. [7]. Alteration of autophagic process was found to be one of the possible reasons of tumor initiation and progression. Indeed, decreased levels of autophagic flux could be correlated to tumor progression, whereas enhanced autophagic flux may be a mechanism supporting tumor survival under hypoxic-, metabolic- or therapeutic-stress conditions. In this context, a better understanding of the stress pathways such as nucleolar and endoplasmic reticulum stress in relation to autophagic process could be useful to investigate novel specific and effective pharmacologic targets for new therapeutic strategies in cancer treatment.

Finally, this collection of review and original research articles summarizes current progress in understanding the molecular mechanisms underlying tumorigenesis and drug resistance, as well as examples of novel therapies based on this new knowledge for treating various types of cancer and overcoming chemoresistance. We believe that these advances will inspire basic and clinical scientists working in cancer treatment-related fields.
